# SUMOylation of Myc-Family Proteins

**DOI:** 10.1371/journal.pone.0091072

**Published:** 2014-03-07

**Authors:** Arianna Sabò, Mirko Doni, Bruno Amati

**Affiliations:** 1 Center for Genomic Science of IIT@SEMM, Fondazione Istituto Italiano di Tecnologia (IIT), Milan, Italy; 2 Department of Experimental Oncology, European Institute of Oncology (IEO), Milan, Italy; Lund University, Sweden

## Abstract

Myc-family proteins are key controllers of the metabolic and proliferative status of the cell, and are subjected to a complex network of regulatory events that guarantee their efficient and fast modulation by extracellular stimuli. Hence, unbalances in regulatory mechanisms leading to altered Myc levels or activities are often reported in cancer cells. Here we show that c- and N-Myc are conjugated to SUMO proteins at conserved lysines in their C-terminal domain. No obvious effects of SUMOylation were detected on bulk N-Myc stability or activities, including the regulation of transcription, proliferation or apoptosis. N-Myc SUMOylation could be induced by cellular stresses, such as heat shock and proteasome inhibition, and in all instances concerned a small fraction of the N-Myc protein. We surmise that, as shown for other substrates, SUMOylation may be part of a quality-control mechanism acting on misfolded Myc proteins.

## Introduction

*Myc*-family genes (c-*myc*, N-*myc* and L-*myc*) are central nodes in developmental, growth-regulatory and oncogenic signaling networks, and are deregulated in a wide range of human cancers (reviewed in [1]). Activation of the *c-myc* proto-oncogene is the leading event in a variety of tumors, such as Burkitt's B-cell lymphomas, in which *c-myc* is translocated near immunoglobulin loci [2], as well as in various carcinomas upon gene amplification [1]. In other cancers, *c-myc* is not structurally altered, although it is frequently over-expressed due to oncogenic activation of upstream signaling pathways (e.g. Ras, Wnt, Notch), and contributes to their growth- and tumor-promoting potential [3]–[5]. Deregulation of N-*myc* has been strongly linked with neural cancers, in particular through gene amplification/overexpression in neuroblastoma, where it is the most consistent marker of poor prognosis and aggressive disease [6]–[9]. The common denominator of the genetic alterations affecting either *myc* gene is the aberrant expression of the corresponding Myc protein. A most illustrating example linking Myc expression levels to cancer incidence is the cancer-associated small nucleotide polymorphism (SNP) rs6983267, which maps to a c-*myc*-regulatory enhancer element in the human genome [10].

c-Myc, N-Myc and L-Myc are related transcription factors of the basic-Helix-Loop-Helix-Leucine Zipper (bHLH-LZ) class, which dimerize with a unique bHLH-LZ protein, Max, to bind E-box consensus motifs in DNA and can have the capacity to either activate or repress transcription (reviewed in [11]). Since alterations in Myc levels potentially lead to unrestrained proliferation and tumorigenesis, it is not surprising that either the proteins or the mRNAs have short half-lives [12]–[14], guaranteeing efficient and fast adaptation of their levels to the status of the cells. As best illustrated for c-Myc, protein turnover is regulated by a complex network of signaling pathways that converge on a set of post-translational modifications, including S62 and T58 phosphorylation [15]. The T58 codon of the translocated c-*myc* allele is frequently mutated in Burkitt lymphoma [16]–[23], resulting not only in reduced interaction with the Fbw7 ubiquitin ligase and increased stability [24], [25], but also reduced pro-apoptotic activity of the c-Myc protein [26]. In *N-myc* amplified neuroblastoma, the high expression of Aurora A, which interacts with both N-Myc and Fbxw7, is required to counteract degradation of N-Myc, thereby uncoupling N-Myc stability from growth factor-dependent signals [27]. In summary, post-translational modifications of the Myc proteins are important in controlling their stability and/or activity, and may hence have an important impact on tumorigenesis. Besides phosphorylation and ubiquitination, c-Myc is also modified by O-linked glycosylation and acetylation [28]. Here, we addressed whether Myc proteins may also be modified by SUMOylation.

SUMOylation is a post-translational modification consisting in the reversible covalent addition of one, or more, small ubiquitin-like modifier (SUMO) proteins. Higher eukaryotes have three SUMO paralogs, SUMO-1, SUMO-2 and SUMO-3 [29]–[31], of which the last two are functionally and structurally very similar [32], [33]. SUMOylation can have three major effects on the target protein. First, since it targets lysine residues, it can compete with other post-translational modifications like acetylation or ubiquitination. Second, it can potentially block protein-protein interactions when occurring in the region mediating the contact. Third, it can create new interaction surfaces on the substrate, in particular through recognition by ligands bearing characteristic SUMO-interacting motifs (SIMs) [34]. Through these biochemical properties, SUMOylation can modulate a variety of biological processes, including transcription, chromosome structure, DNA repair, macromolecular assembly, trafficking, or signal transduction (reviewed in [35]–[37]). Logically, therefore, unbalances in SUMOylation/deSUMOylation cycles may underlie a variety of diseases, including cancer [38].

In contrast with the complexity of the Ubiquitination machinary, SUMOylation is mediated by few enzymes that act on all the target proteins: a unique E1 SUMO-activating enzyme (the SAE1/2 complex), a unique E2 SUMO-conjugating enzyme (Ubc9) and a limited series of E3 ligases [39]. Mutations or inhibition of the E1 or E2 enzymes, may thus affect the SUMOylation of all the target proteins at once, making it difficult to dissect the functions of this modification on specific substrates. As an example, it has recently been shown that the SAE1 gene is a transcriptional target of Myc [40] and that SAE1/2 activity is required to sustain the growth of Myc dependent tumors in mice [41], but whether this is related to direct SUMOylation of Myc itself has not been addressed. In a different context, human fibroblasts, SUMOylation has been reported to repress transcription of genes associated with cell growth and proliferation [42].

We report here that Myc proteins, in particular c- and N-Myc, are SUMOylated on a lysine positioned between the conserved Myc-Box IV and bHLH-LZ domains. This modification is induced by protein-damaging stresses or proteasome inhibition. So far, no evident effect of SUMOylation on Myc activity has emerged from a series of functional assays. We speculate that this modification may constitute a quality control mark for misfolded Myc proteins.

## Materials and Methods

### Cell culture, transfection and reagents

293T, U2OS, HeLa and SK-N-AS cells were cultivated in DMEM medium supplemented with Glutamine, 10% FBS, 100 units/ml Penicillin and 100 mg/ml Streptomycin (hereafter “antibiotics”). MEFs were cultivated in DMEM medium supplemented with Glutamine, 10% FBS, antibiotics, 0.1 mM Non-Essential Amino Acids (hereafter NEAA) and βmercaptoethanol. SHSY5Y and SK-N-BE(2) were cultivated in MEM:HAM'S F12 (1∶1) medium supplemented with Glutamine, 15% FBS, antibiotics and NEAA. SK-N-BE/2C were cultivated in RPMI 1640 medium supplemented with 10% FBS, Glutamine, antibiotics, Sodium Pyruvate (hereafter NaP) and NEAA. SIMA and Kelly were cultivated in RPMI 1640 supplemented with 10% FBS, antibiotics and Glutamine. IMR32 cells were cultivated in MEM medium supplemented with 10% FBS, antibiotics, NEAA, NaP and βmercaptoethanol. Cells were transfected using Lipofectamine 2000 (LifeTechologies) following manufacturer instructions, except for 293T cells, which were transfected with the calcium phosphate method: in co-transfection experiments a total of 5 µg (with Lipofectamine 2000) or 10 µg (with calcium phosphate) of DNA were used, with a ratio of 1∶1 between Flag-tagged N-Myc and HA-tagged SUMO-1 or -2. All co-transfections were balanced with the corresponding empty vectors to keep total amount of transfected DNA constant. Cells were treated with 10 µM MG132 (Sigma) or DMSO as a control for 6 h where indicated. Cycloheximide (Sigma) was used at 50 µg/ml for the indicated times.

### Antibodies

The following antibodies were used for immunoblotting: FLAG DDDDK (ab1162, Abcam), Flag M2 (Sigma), HA (HA.11, Covance), SUMO-1 S8070 (Sigma), SUMO-2 (ab22654, Abcam), N-myc (2) (sc-142, Santa Cruz), N-Myc M50 (sc-22836, Santa Cruz), Vinculin (V9264, Sigma), p19-ARF 5-C3-1 (sc-32748, Santa Cruz) Anti-phospho-Histone H2A.X (Ser 139, Millipore); p53 (1C12, Cell Signaling). For immunoprecipitation N-Myc OP13 (NCM II 100, Calbiochem) was used.

### Plasmids

pcDNA3 HA-SUMO-1 and HA-SUMO-2 were kindly supplied by Susanna Chiocca. CβF-Flag c-, N-, L-Myc (mouse cDNAs) were kindly supplied by Steven McMahon. The Myc point mutants were generated using site-directed mutagenesis by PCR, subcloned in a pCDNA3-Flag or pCMV-Flag-DEST vector, and confirmed by DNA sequencing. The pBabePuro N-MycER™ vector (WT and K349R) was created by PCR amplification of a mouse N-Myc cDNA, digestion with EcoRI/BamHI and co-ligation of the fragment in the pBPΔCla vector (EcoRI/SalI) together with the ER cDNA moiety obtained by digestion (BamHI/SalI) from the pBP-MycER vector (kind kift of T. Littlewood). pQCXIP and pQCXIN Flag-HA-N-Myc (WT and K349R) vectors were created by subcloning FLAG-HA-N-Myc from pBabePuro-Flag-HA vector (in which the cDNA of N-Myc was previously subcloned from the corresponding pCDNA3-Flag vector) in pQCXIP and pQCXIN (Clontech). The pGIPZ lentiviral vector (V2LHS_36755) encoding an shRNA against human N-myc was purchased from Open Biosystems.

### Viral infection

High-titer retroviral and lentiviral supernatants were obtained by transfecting Phoenix or 293T cells respectively with the corresponding vectors. Targets cells were subjected to three cycles of infection, 24 h of recovery and selection with the appropriate antibiotic (puromycin or neomycin) for 3 days. pBabePuro-N-MycER cells where treated with 400 nM 4-hydroxy-tamoxifen (4-OHT, Sigma) or ethanol as control, as indicated.

### RNA extraction and analysis

Total RNA was purified onto RNeasy columns (Qiagen) and treated on-column with DNase (Qiagen). Complementary DNA (cDNA) was produced using the reverse-transcriptase ImPromII (Promega). 10 ng of cDNA were used for Real-time PCR reactions with FAST SYBR Green Master Mix (Applied Biosystems). Primers for mRNA analysis were designed by using computer assisted primer design software (Primer3). The sequences of primers used in this study are the following:

mouse CAD (fw AP13: CGGGCCAAGAAGAAGATGG,

rv AP14: GGTCCGAGTCCACCTCCAC);

mouse Car12 (fw AP10505: TCCTACCCCCAGAGAAATGA,

rv AP10506: GGGCCACTGAAAGGATGATA);

mouse St6 (St6galnac4) (fw AP10507: TGGTCTACGGGATGGTCA,

rv AP10508: CTGCTCATGCAAACGGTACAT);

mouse Reep6 (fw AP10509: GTGCAATGTCATCGGATTTG,

rv AP10510: TTGCCCGCGTAGTAGAAAG);

mouse Nucleolin (fw AP1985: GTCTGAGGATACCACTGAAG,

rv AP1986: GCCCAGTCCAAGGTAACT);

human Nucleolin (fw AP387: AGAGCAATCAGGCTGGAGTTG,

rv AP388: TTCAGTGGTATCCTCAGACAGGC);

human CAD (fw AP547: GACCATGAGCTGCTATGC,

rv AP548: GGATGGTGAAGATGTCCAG);

human IFRD2 (fw AP385: CCGTAAGGGCAACACGCT,

rv AP386: GTGCTGCGGGCCTCACT);

human ID2 (fw AP 2669: AACAGCCTGTCGGACCAC,

rv AP2670: CTTGGAGTAGCAGTCGTTC);

human and mouse RPPO (fw AP1209: TTCATTGTGGGAGCAGAC,

rv AP1210: CAGCAGTTTCTCCAGAGC).

### Immunoprecipitation/Immunoblot analysis

For immunoblot and immunoprecipitation in denaturing conditions, cells were lysed in SDS lysis buffer made by 1∶3 ratio of Buffer I (5% SDS, 0.15 M Tris-HCl pH 6.8, 30% glycerol) and Buffer II (25 mM Tris-HCl pH 8.3, 50 mM NaCl, 0.5% NP-40, 0.5% deoxycolate, 0.1% SDS, 1 mM EDTA) supplemented with protease inhibitors cocktail (Roche), 1 mM DTT and 5 mM N-Ethylmaleimide (NEM, Sigma) and denatured 5 min at 95°C. Cell lysates were then sonicated and centrifuged at maximum speed for 10 min. Supernatants were either directly resolved by SDS-PAGE or diluted 1∶5 in E1A buffer (50 mM Hepes pH 7.5, 250 mM NaCl, 0.1% NP-40, 1 mM EDTA, supplemented with protease inhibitors cocktail, 1 mM DTT and 5 mM NEM) and then immunoprecipitated using anti anti-Flag M1 beads (Sigma) or anti-N-MYC antibodies and Protein G beads (Zymed). Co-immunoprecipitation was carried out with a co-IP lysis buffer containing 50 mM Tris pH 8, 5 mM EDTA, 150 mM NaCl, 0.5% NP-40, supplemented with protease inhibitors cocktail (Roche), 5 mM NEM and 20 mM iodoacetamide (IAA, Sigma). Cells were rinsed with ice-cold PBS and lysed on ice for 30 min in lysis buffer, sonicated and centrifuged at maximum speed for 10 min. Supernatants were immunoprecipitated using anti anti-Flag M1 beads. Immunoprecipitates were resolved by SDS-PAGE, transferred to a nitrocellulose membrane (Whatman), and blotted using the antibodies indicated in the figures. Detection was performed using ECL (Amersham). For quantitative immunoblotting, membranes were incubated with IRDye secondary antibodies and analyzed through the Odissey System (Li-cor).

### Proliferation, cell cycle and apoptosis

BrdU incorporation was analyzed as described [43]. Cell death in MEFs cultures was assessed with the Caspase-Glo 3/7 kit (Promega), while cell growth was monitored using the CellTiter-Glo Luminescent Cell Viability Assay (Promega).

### Dual luciferase assay

HeLa cells were transfected with Lipofectamine 2000 (LifeTechnologies) with 50 ng of pNuc-Luc or p15-Luc reporter, 500 ng of pCMV-Flag-Myc (c-Myc, N-Myc WT or N-Myc K349R). For normalization and transfection efficiency control we used 10 ng of pRL-TK reporter (Promega) that constitutively expresses the Renilla luciferase. After 48 hours cells were lysed and assayed for luciferase activity using the Dual Luciferase kit (Promega).

## Results and Discussion

### N-Myc is modified by SUMO-1 and SUMO-2 at lysine 349

**Figure 1:**
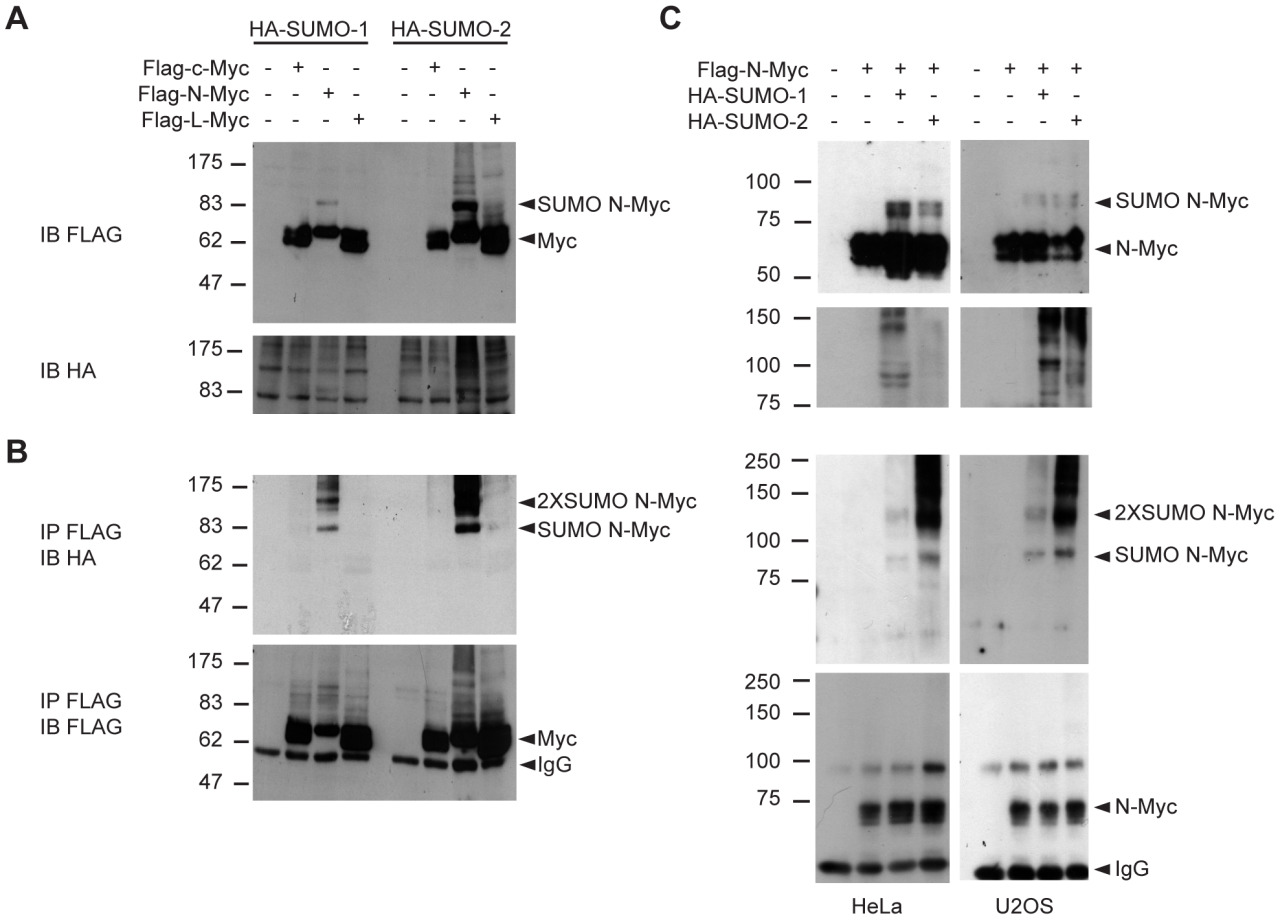
SUMOylation of Myc proteins. (**A**) Immunoblot analysis in denaturing conditions of 293T cells transfected with plasmids expressing the indicated proteins. (**B**) Lysates from (**A**) were immunoprecipitated (IP) in denaturing conditions with anti-Flag beads, and the precipitates subsequently analyzed by immunoblotting (IB) with the indicated antibodies. (**C**) as in (**A**) and (**B**) but for HeLa and U2OS cells.

Co-expression of Flag-tagged c-, N-, or L-Myc and HA-tagged SUMO-1 or SUMO-2 in 293T cells followed by lysis in denaturing conditions and immunoblot with anti-Flag antibodies led to the appearance of a secondary N-Myc band with a molecular weight compatible with the addition of a SUMO molecule in the cells co-expressing N-Myc and either SUMO-1 or SUMO-2 (“SUMO N-Myc”, Fig. 1A). Immunoprecipitation of the Flag-Myc proteins with anti-Flag antibodies under denaturing conditions followed by immunoblotting with anti HA antibodies yielded a band of the same molecular weight, showing that this corresponded to N-Myc conjugated with one SUMO molecule (Fig. 1B). A slower migrating band was also detected, most likely corresponding to the addition of two copies of SUMO per N-Myc molecule. We confirmed this result in two different cell lines, HeLa and U2OS, in which we also verified that the signal we obtained by immunoblotting with the anti-HA antibody was specific, as this was absent in cells that overexpressed only Flag-N-Myc but not HA-tagged SUMO proteins (Fig. 1C). In all three cell lines, blotting of N-Myc immunoprecipitates with anti-HA antibodies also yielded smears of very high molecular weight, suggesting addition of more SUMO proteins or of a combination of SUMO and ubiquitin proteins [44] to a single N-Myc polypeptide.

**Figure 2:**
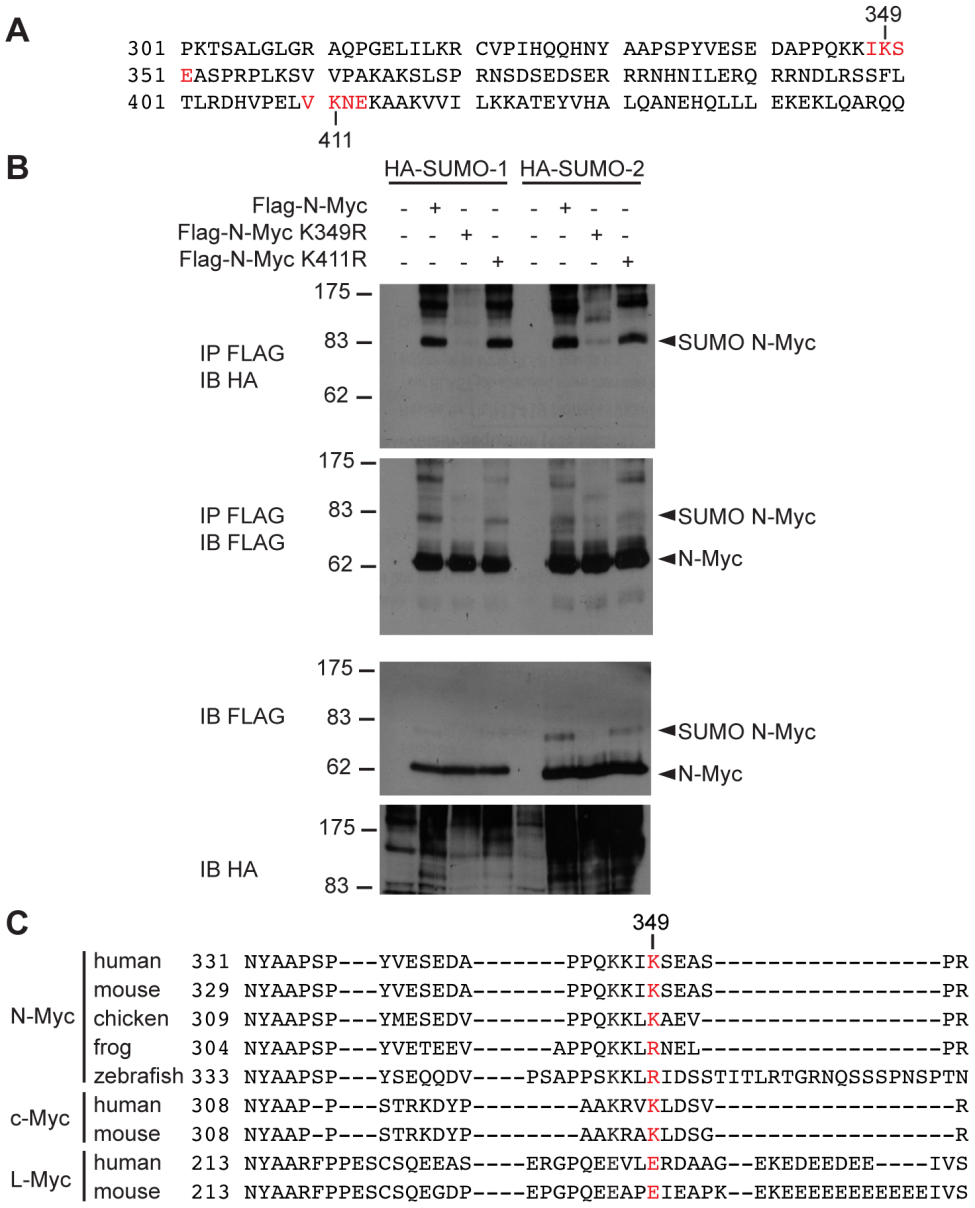
N-Myc is SUMOylated at lysine 349. (**A**) Mouse N-Myc protein sequence (from aa 301 to 450) with consensus SUMO acceptor sites (as predicted by SUMOplot™) highlighted in red. (**B**) 293T cells were transfected with plasmids expressing the indicated proteins. Lysates were analyzed by immunoprecipitation and immunoblotting as indicated in Fig. 1. (**C**) The Myc region corresponding to the SUMO acceptor site in mouse N-Myc is aligned with N-Myc sequences from other species and also c-Myc and L-Myc corresponding regions. Mouse N-Myc lysine 349 is highlighted in red.

**Figure 3:**
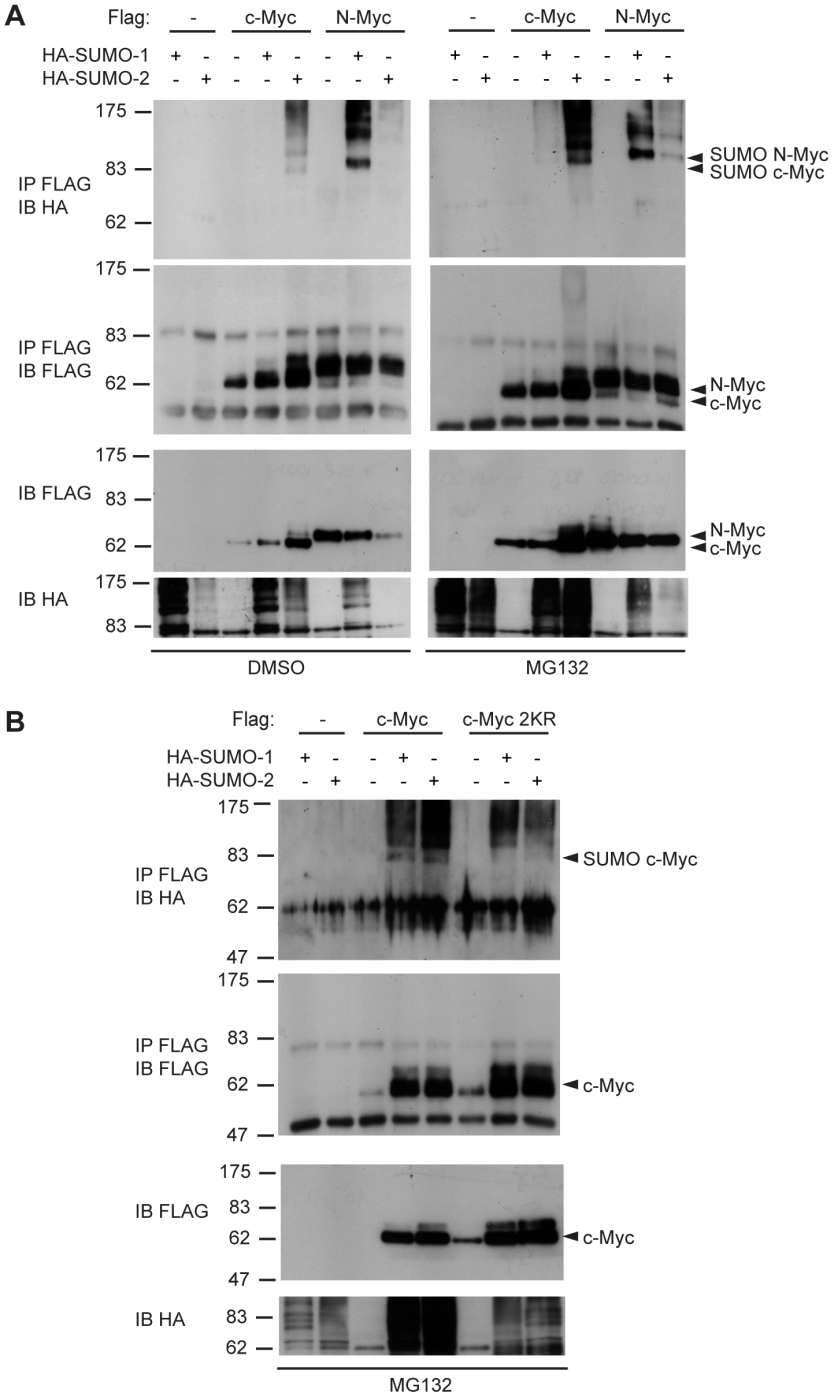
SUMOylation of c-Myc at lysines 323 and 326. (**A**) 293T cells were transfected with plasmids expressing the indicated proteins. Lysates were analyzed by immunoprecipitation and immunoblotting as indicated in Fig. 1. Cells were treated with MG132 10 µM or DMSO for 6 h, as indicated. (**B**) as in (**A**) but with wild type or K323, 326R (2KR) mutant c-Myc proteins.

By exploiting the SUMOplot™ program (http://www.abgent.com/sumoplot), we identified two putative SUMO consensus sites corresponding to lysines 349 and 411 in the mouse N-Myc coding sequence (Fig. 2A). We mutated either of those residues to arginine to obtain single point mutant Flag-N-Myc expression vectors that we used to test conjugation of SUMO-1 or SUMO-2 in co-transfected 293T cells. While N-Myc K411R was modified at a level comparable to the wild type protein, the K349R mutation abolished N-Myc SUMOylation (Fig. 2B). We conclude that the main SUMO-acceptor site on mouse N-Myc is K349, a residue that is conserved in human and chicken N-Myc but not in more distant species such as frog and zebrafish (Fig. 2C). Interestingly, c-Myc has a slightly different SUMO consensus site in the corresponding region (K326, Fig. 2C), while L-Myc lacks a clear consensus. Indeed, whereas in most experiments only N-Myc appeared to be SUMO-conjugated, we could also sometimes detect c-Myc SUMOylation, in particular following treatment of the cells with the proteasome inhibitor MG132 (Fig. 3A). Furthermore, a double c-Myc mutant lacking K323 and K326 (c-Myc 2KR) appeared to lose the main SUMO acceptor site (Fig. 3B); of note, high molecular weight smears of SUMO-conjugated c-Myc were still detectable, which may indicate SUMOylation of other sites, or the incorporation of SUMO moieties into poly-SUMO and/or poly-ubiquitin chains [44]. We conclude that the main SUMO-acceptor sites are K349 on N-Myc and most likely the corresponding K326 on c-Myc (or the adjacent K323; Fig. 2C). These residues lie between the conserved element Myc box IV [45] and the C-terminal basic-helix-loop-helix (bHLH-LZ) motif, essential for dimerization with Max and DNA binding [46]–[48]. We focus hereafter on N-Myc SUMOylation, which was the most consistently observed in our experiments.

### Loss of SUMOylation does not reveal alterations in N-Myc biological activities

**Figure 4:**
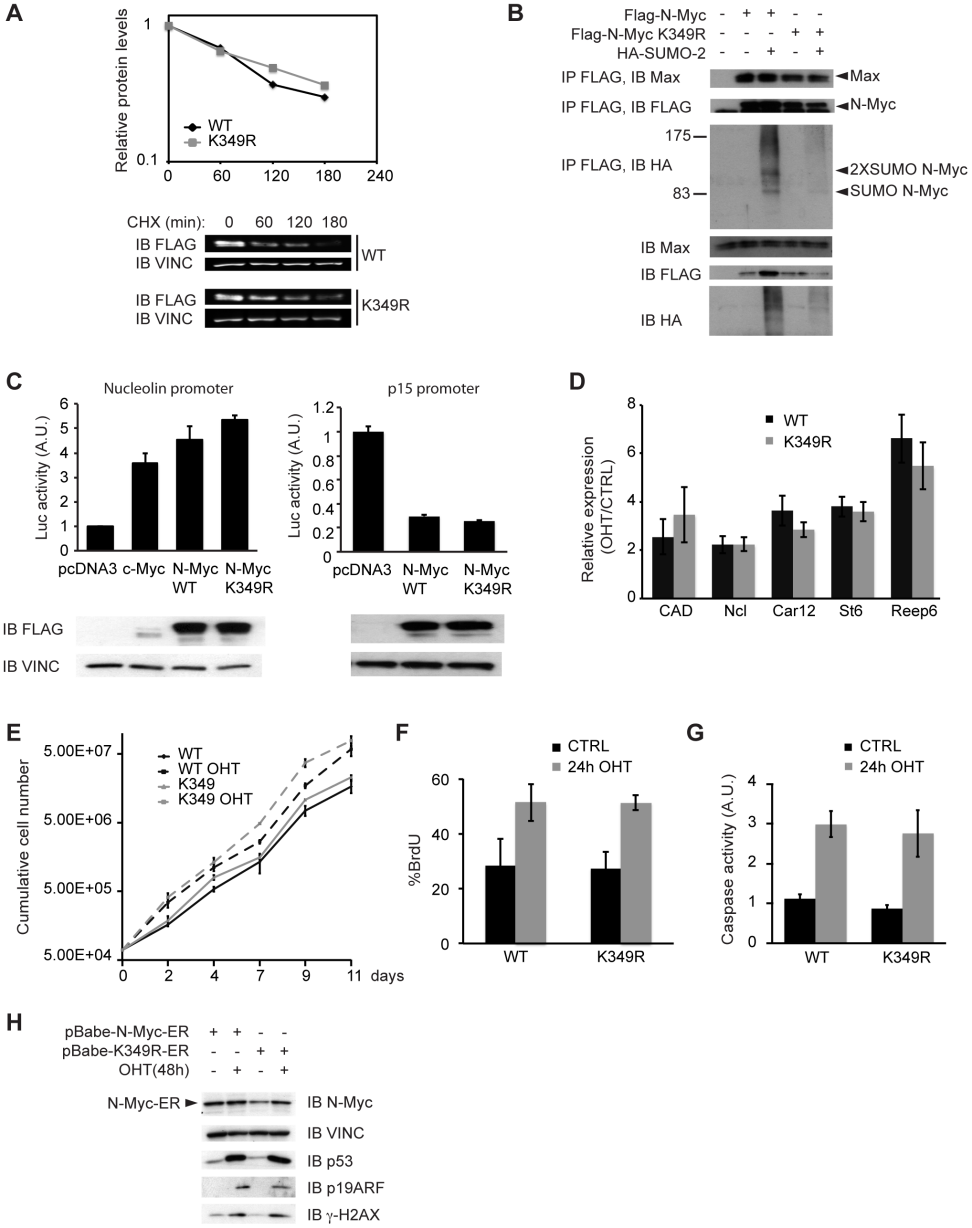
SUMOylation defective N-Myc mutant does not reveal critical differences respect to the wild type counterpart. (**A**) U2OS cells transfected with Flag tagged N-Myc WT or K349R mutant were treated with CHX 50 µg/ml for the indicated times. Cells were then lysed and the levels of the Flag-N-Myc protein were measured by quantitative immunoblotting. The graph represents the means of three independent experiments. Immunoblot of one representative experiment is shown. It is noteworthy that the half-life of exogenous N-Myc measured here (ca. 110 min) is in range with that seen in analogous experiments for either N-Myc (157 min [27]) or c-Myc (97-100 min [54], [65]). (**B**) 293T cells were transfected with plasmids expressing the indicated proteins. Lysates were analyzed by immunoprecipitation and immunoblotting as indicated in Fig. 1, but in non-denaturing conditions (co-IP lysis buffer: see methods). (**C**) HeLa cells were transfected with the reporter plasmids pNuc-Luc (left panel) or p15-Luc (right panel) together with pRL-TK (as a normalizer) and expression plasmids for Flag-c-Myc, Flag-N-Myc WT or Flag-N-Myc K349R, as indicated. The transcriptional activity was measured with a Dual Luciferase Assay kit (Promega). The histograms represent the mean and s.d. of three independent experiments. The total levels of the indicated proteins were assessed by immunoblot. (**D-H**) Primary MEFs were infected with retroviral vectors coding for N-MycER™ WT or K349R and treated or not with 4-hydroxy-tamoxifen (OHT). (**D**) RT-PCR measurement of target mRNAs in OHT treated (48 h) versus control cells after normalization to the housekeeper RPPO. The histogram represents the mean and s.d. of three independent experiments. (**E**) Growth curves showing cumulative cell numbers for N-MycER™ WT or K349R MEFs treated or not with OHT for up to 11 days. The curves represent the mean and s.d. of three independent cell counts. (**F**) FACS analysis of BrdU incorporation (% of positive cells) after 24 h of OHT or control treatment. The histogram represents the mean and s.d. of three independent experiments. (**G**) Luminescence-based measurement of Caspase activity (Caspase-Glo 3/7, Promega) after 24 h of OHT or control treatment, the bars represent the mean and s.d. of three independent experiments. In neither of the quantitative assays used (panels A, C, D, E, F, G) was a statistically significant difference observed between the WT and K349R forms of N-Myc. (**H**) Immunoblot analysis of p53, ARF and γH2AX after 48 of OHT or control treatment.

On many substrates, SUMOylation can crosstalk with the ubiquitin-proteasome system (reviewed in [49], [50]). The K349 residue in N-Myc lies between MBIV and the bHLH-LZ, a region that was previously associated to Myc ubiquitination and acetylation with possible effects on protein stability [51]–[54]. To address whether K349 SUMOylation may modulate N-Myc stability, we transfected U2OS cells with Flag tagged N-Myc (WT or K349R mutant), treated the cells with CHX to block protein synthesis for 60, 120 or 180 min, and measured the residual Flag-N-Myc protein by quantitative immunoblotting. The wild-type and mutant proteins showed identical decay rates, suggesting that SUMOylation does not regulate bulk N-Myc degradation (Fig. 4A).

We then compared the binding of WT and K349R N-Myc to Max in 293T cells. Both N-Myc proteins, could co-immunoprecipate endogenous Max either with or without co-transfection of a SUMO-2 expression vector (which induced SUMOylation of WT N-Myc), providing no indication that SUMOylation regulates N-Myc/Max dimerization (Fig. 4B). To further investigate possible effects of SUMOylation on N-Myc transcriptional activity, we performed luciferase reporter assays in HeLa cells with reporter constructs driven either by a Myc-activated or a Myc-repressed promoter (i.e. Nucleolin and p15): no significant differences between the activities of WT and K349R N-Myc were revealed in either assay (Fig. 4C). To address the regulation of endogenous Myc-target genes, we infected primary MEFs with retroviral vectors encoding WT or K349R N-Myc fused to the hormone binding domain of the Estrogen Receptor (N-MycER™), allowing conditional activation of N-Myc by 4-hydroxy-tamoxifen (OHT) [55]. RT-PCR measurement of target mRNAs revealed no significant effect of the K349R mutation on N-Myc transcriptional activity (Fig. 4D).

We exploited the N-MycER™ expressing MEFs to evaluate whether SUMOylation of N-Myc had any role in N-Myc-dependent proliferation or apoptosis. Activation of N-MycER™ induced a slight increase in overall growth rates, but the WT and K349R proteins showed no difference in this regard (Fig. 4E). Both proteins were also comparable in their ability to enhance the percentages of cells in S-phase (Fig. 4F) or to trigger apoptosis (Fig. 4G). In addition western blot analysis showed that both proteins induced a similar activation of the ARF/p53 pathway and of the DNA Damage Response (Fig. 4H). In summary, we were unable to reveal any obvious effects of SUMOylation on any of the Myc activities tested (dimerization, transcription, proliferation, apoptosis), albeit our data do not allow us to rule out more subtle effects. Indeed, SUMOylation of a given substrate is typically transient and occurs on a small fraction of the protein, as consistently observed here (in our experiments, the intensity of the bands corresponding to SUMOylated N-Myc is at most 10–20% of that of the unmodified form), making it quite difficult to pinpoint any functional role of this post translational modification (reviewed in [56]). Hence, while our data with the K349R mutant rule out any essential role of SUMOylation in N-Myc activity, an altered function of SUMO-conjugated N-Myc remains plausible.

### Endogenous N-Myc is SUMOylated in neuroblastoma cells

**Figure 5:**
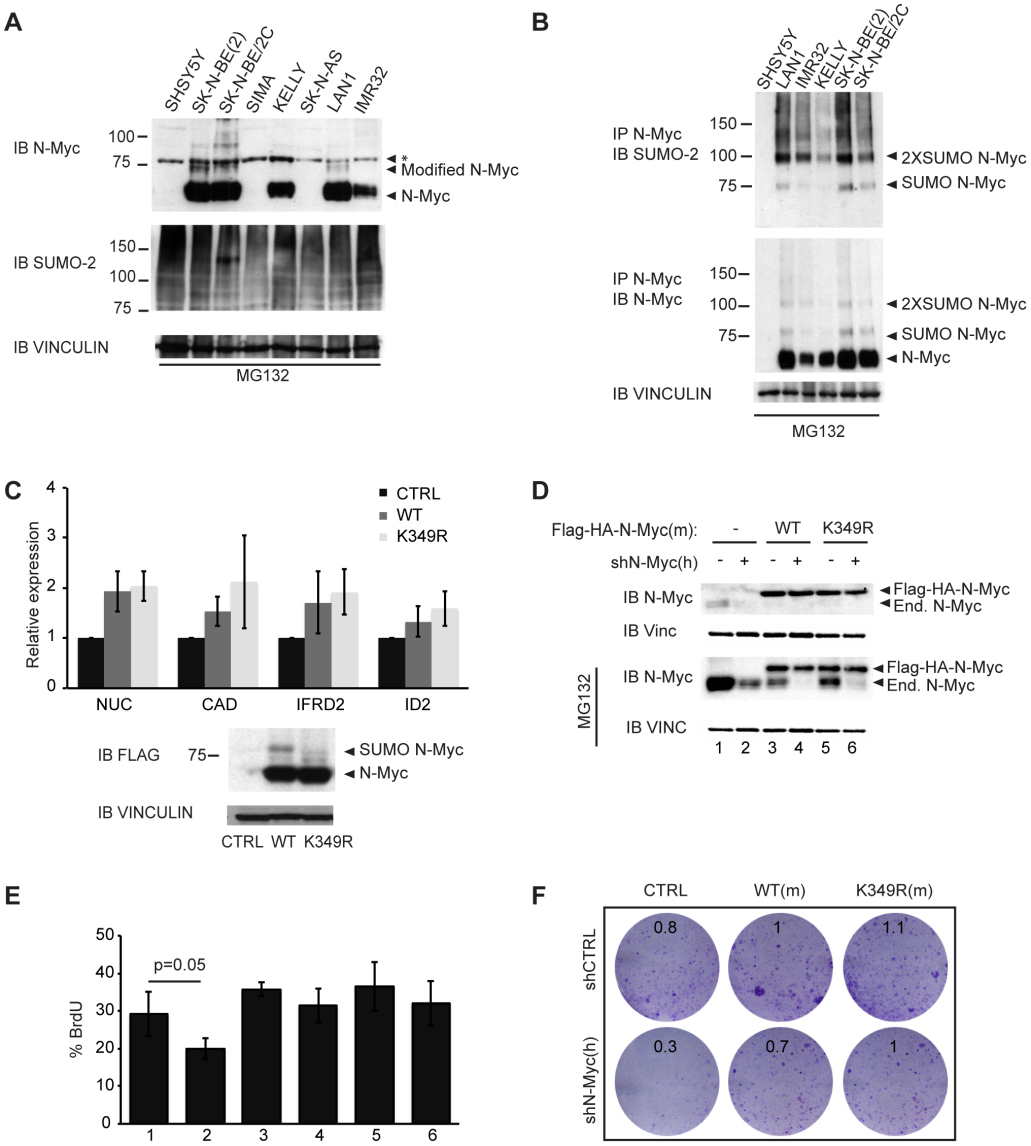
SUMOylation of N-Myc in neuroblastoma cells. (**A**) Immunoblot analysis in denaturing conditions of different neuroblastoma cell lines after 6 h treatment with 10 µM MG132. (*): non-specific band. (**B**) Immunoprecipitation (IP) with anti-N-Myc antibodies in denaturing conditions and analysis by immunoblotting with the indicated antibodies. (**C**) SHSY5Y cells were infected with pQCXIP retroviral vectors expressing Flag-HA tagged WT or K349R N-Myc proteins and expression of selected mRNAs was measured by RT-PCR (after normalization to the housekeeper RPPO). The histogram represents the mean and s.d. of three independent experiments. The total levels of the indicated proteins were assessed by immunoblot. (**D-F**) SK-N-BE(2) cells were infected with pQCXIN empty vector, Flag-HA-N-Myc WT or K349R (mouse cDNA) and superinfected with pGIPZ-PURO shN-Myc or control shRNA. (**D**) Immunoblot analysis of the level of endogenous and Flag-HA tagged exogenous N-Myc protein in mock treated cells and cells treated with MG132 10 µM. (**E**) FACS analysis of BrdU incorporation (% of positive cells); the histogram represents the mean and s.d. of three independent experiments. The numbering of the samples corresponds to that in (**D**). In neither of the quantitative assays used (panels C, E) was a statistically significant difference observed between the WT and K349R forms of N-Myc. (**F**) Colony Assay. Cells were plated in 6-well plates, incubated for 7 days and stained with Crystal violet. Numbers in each plate indicate relative cell densities, as assessed by absorbance at 595 nm following solubilization of the dye with acetic acid.

Amplification of the *N-myc* gene in neuroblastoma leads to high N-Myc protein levels. Immunoblot analysis of neuroblastoma cell lines treated with the proteasome inhibitor MG132 (inducing further N-Myc accumulation) revealed the appearance of a faint slower migrating band above the main N-Myc signal (Fig. 5A, “Modified N-Myc”). The increase in molecular weight (ca. 15 kDa) was compatible with the addition of either an ubiquitin or a SUMO molecule. We thus subjected MG132-treated neuroblastoma cell lysates to immunoprecipitation in denaturing conditions with an anti-N-Myc antibody followed by immunoblot with an anti-SUMO-2 antibody, demonstrating that the higher molecular weight bands corresponded to SUMOylated N-Myc (Fig. 5B).

As SHSY5Y neuroblastoma cells do not express endogenous N-Myc (Fig. 5A), we infected those cells with retroviral vectors expressing WT or K349R N-Myc. While a band consistent with SUMOylation was detected with WT N-Myc, this was absent with the K349R mutant (Fig. 5C, bottom). Consistent with the functional data presented above, we observed no significant difference in the transcriptional induction of target genes by either of the two forms of N-Myc (Fig. 5C, top).

A number of experimental strategies suggest that N-*myc*-amplified neuroblastoma cells are addicted to high levels of N-Myc, at least in tissue culture [27], [57]–[60]. We thus decided to functionally compare the capacity of exogenously expressed WT and K349R N-Myc proteins to rescue cell growth in SK-N-BE(2) neuroblastoma cells depleted of the endogenous N-Myc protein by RNA interference. The cells were infected with retroviral vectors expressing WT or K349R Flag-HA tagged mouse N-Myc proteins, or control empty vector, and super-infected with a lentiviral vector expressing either an shRNA targeting human N-Myc or a control shRNA. Immunoblot analysis confirmed both the effective knockdown of endogenous N-Myc and the over-expression of tagged WT or K349R N-Myc (Fig. 5D). We then measured the percentage of cells in S-phase through BrdU incorporation revealing that the knock down of endogenous N-Myc led to a slight decrease in proliferation [58], [61] that could be compensated by either WT or K349R N-Myc (Fig. 5E). A similar result was obtained with a colony assay (Fig. 5F). As above, these data allow us to rule out an essential role of SUMOylation in the growth-regulatory activity of N-Myc in neuroblastoma cells, albeit it remains to be addressed whether the small fraction of SUMO-conjugated N-Myc may be functionally altered.

### N-Myc SUMOylation is induced by cellular stresses, in particular heat shock

**Figure 6:**
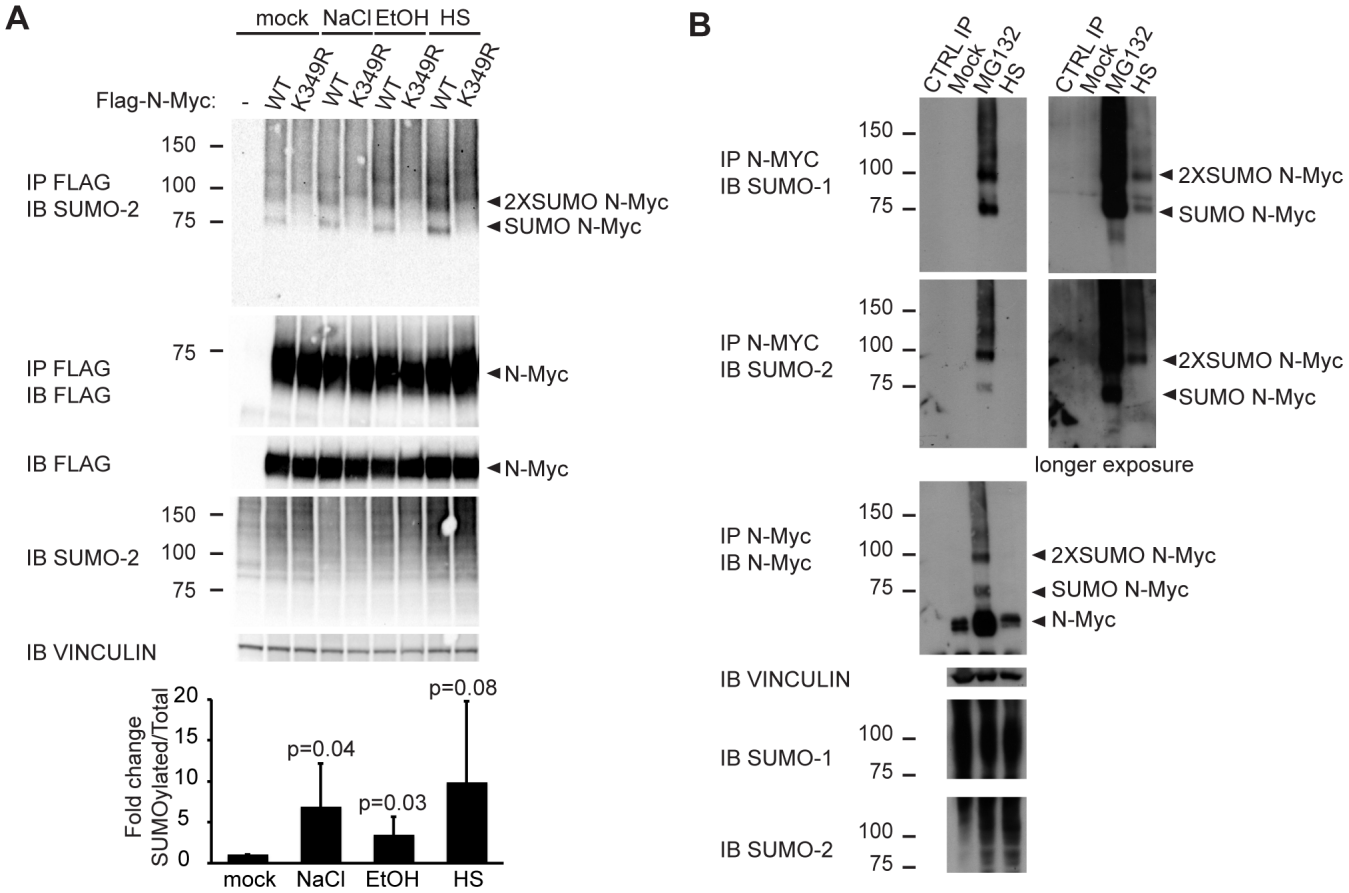
Protein stresses induce N-Myc SUMOylation. (**A**) HeLa cells were transfected with plasmids expressing the indicated proteins and either mock treated or treated for 30 min with 0.7 M NaCl, 3.7% EtOH or heat-shock at 43°C (HS). Lysates were analyzed by immunoprecipitation and immunoblotting in denaturing conditions with the indicated antibodies. The histogram at the bottom represents the quantification of the ratio of monoSUMOylated to total wild-type N-Myc, normalized to the mock-treated cells: values represent the mean and s.d. from five independent experiments. (**B**) Lan-1 cells were either mock treated, treated with 10 µM MG132 for 6 h or heat-shocked at 43°C for 1 h (HS). Lysates were analyzed as in **A**. CTRL IP: IP with N-Myc antibody without cell lysate.

Protein-damaging stresses (heat shock, oxidative stress, ethanol addition, osmotic stress) have been reported to stimulate SUMO conjugation to several targets [32]. Indeed, in transfected HeLa cells, SUMOylation of N-Myc was induced (although at variable levels in different experiments) by exposure of the cells to either NaCl, ethanol or heat shock (Fig. 6A). Proteomic analysis of the global response of SUMO-2/3 to heat shock revealed changes in the SUMOylation status of a large set of proteins (among which also c-Myc itself) [62] that was shown to be similar to that seen after long-term inhibition of the proteasome with MG132 [63], leading to the hypothesis that the accumulation of misfolded proteins occurring in both conditions is the signal that triggers SUMO-2 conjugation, SUMO acting here mainly as a quality control mark for protein folding [63], [64]. Consistent with this idea, endogenous N-Myc was SUMOylated in Lan1 neuroblastoma cells upon either heat shock or treatment with the proteasome inhibitor MG132 (Fig. 6B).

In summary, we have detected SUMOylation of a small fraction of N-Myc upon either overexpression, heat shock treatment or proteasome inhibition. Combined with the apparent lack of a functional effect of SUMOylation on the bulk of N-Myc activities, the most likely interpretation of our data is that this modification occurs selectively on the inactive/misfolded pool of N-Myc. It remains to be addressed whether these SUMOylated N-Myc molecules are then destined to a controlled re-folding process or, alternatively, to selective degradation. In either case, SUMO would provide an important quality control system, preventing the accumulation of altered Myc proteins.
